# Formation of Ga droplets on patterned GaAs (100) by molecular beam epitaxy

**DOI:** 10.1186/1556-276X-7-550

**Published:** 2012-10-03

**Authors:** Ming-Yu Li, Yusuke Hirono, Sabina D Koukourinkova, Mao Sui, Sangmin Song, Eun-Soo Kim, Jihoon Lee, Gregory J Salamo

**Affiliations:** 1 College of Electronics and Information, Kwangwoon University, Nowon-gu Seoul 139-701, South Korea; 2 Institute of Nanoscale Science and Engineering, University of Arkansas, Fayetteville 72701, AR, USA

## Abstract

In this paper, the formation of Ga droplets on photo-lithographically patterned GaAs (100) and the control of the size and density of Ga droplets by droplet epitaxy using molecular beam epitaxy are demonstrated. In extension of our previous result from the journal *Physical Status Solidi A*, volume 209 in 2012, the sharp contrast of the size and density of Ga droplets is clearly observed by high-resolution scanning electron microscope, atomic force microscope, and energy dispersive X-ray spectrometry. Also, additional monolayer (ML) coverage is added to strength the result. The density of droplets is an order of magnitude higher on the trench area (etched area), while the size of droplets is much larger on the strip top area (un-etched area). A systematic variation of ML coverage results in an establishment of the control of size and density of Ga droplets. The cross-sectional line profile analysis and root mean square roughness analysis show that the trench area (etched area) is approximately six times rougher. The atomic surface roughness is suggested to be the main cause of the sharp contrast of the size and density of Ga droplets and is discussed in terms of surface diffusion.

## Background

In the last two decades, a number of semiconductor quantum and nanostructures (QNSs) by the strain-driven self-assembly based on Stranski-Krastanow (S-K) growth [1] have been demonstrated in the field of epitaxial growth using molecular beam epitaxy (MBE). As a result, various device applications have been demonstrated such as lasers, detectors, sensors, photovoltaic cells, light-emitting diodes, and solid-state quantum computation [2-7]. Meanwhile, droplet epitaxy (D-E) proposed by Koguchi et al. in 1991 [8] has been relatively recently gaining increased interests due to its advantages over the conventional S-K growth approach for the fabrication of low-dimensional epitaxial semiconductor QNSs [9-23]. While the strain induced by the lattice mismatch is required in the S-K approach, it is not essential in the D-E approach for the fabrication of epitaxial QNSs. As a result, the selection of material system for QNSs by D-E approach is highly elastic and thus, a variety of unseen configurations of epitaxial QNSs have been demonstrated by D-E approach [9-23]. In addition, not only D-E approach can be used for lattice matched systems but also can be applied in the lattice mismatched systems. Quantum dots (QDs) and quantum rings are the most commonly studied epitaxial QNSs [9-14]. QD molecules [15-19], low-density QDs [20], ensembles of quantum ring geometry and droplet [21], and various nanostructure complexes [22,23] have been demonstrated by the D-E approach. In addition, nanohole drilling and local etching effect [24-26], selective etching using droplet as a mask [27,28], various configurations of In nanocrystals [29,30], running droplets [31-33], and Ga-triggered oxide desorption [34,35] are only a few examples of D-E applications.

The fabrication of epitaxial QNSs is inherently dependent on the size, shape, and density of initial liquid phase metal droplets (MDs) and consequently, the control of the density and size of MDs becomes an essential research focus. The control of droplets on planar substrates has been somewhat widely studied [9-23,36,37]; however, the fabrication of MDs on patterned surfaces lacks its investigation. This very naturally puts the control of MDs on patterned substrate as an attractive and essential research topic. In this paper, therefore, in extension of our previous results [38,39], we extend the results of the sharp contrast of the size and density of Ga MDs on photo-lithographically patterned GaAs (100) by D-E approach using MBE. As evidenced by 3-D atomic force microscope (AFM) and high-resolution scanning electron microscope (SEM), the sharp contrast of the size and density of Ga MDs is clearly observed, showing an order magnitude higher density on the trench area (the etched area). Conversely, the size is much larger on strip top area (the un-etched area). By systematically varying the monolayer (ML) coverage, we demonstrate the control of size and density of Ga MDs on patterned GaAs (100) surface. The atomic surface roughness is around six times higher on the trench area (etched area) based on the cross-sectional line profile and root mean square (RMS) roughness analysis. The sharp contrast of size and density of Ga MDs is discussed in terms of surface adatom diffusion.

## Methods

### Experimental details

**Figure 1:**
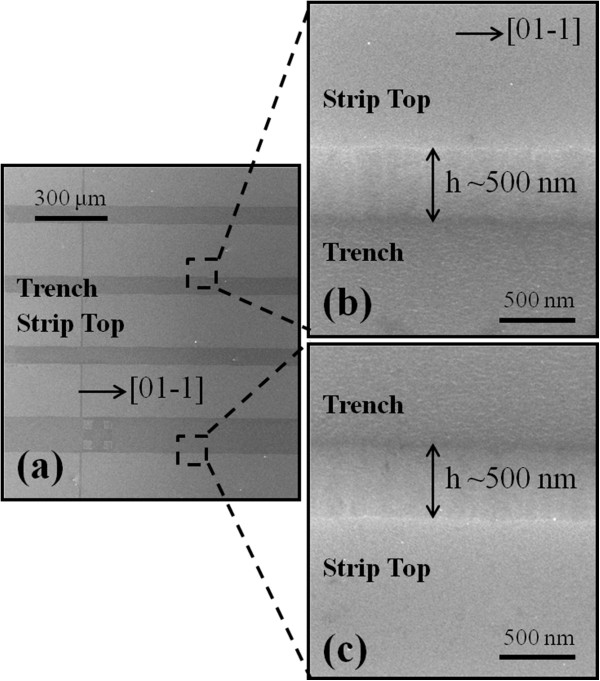
**SEM images of interface between trench(patterned)and strip top(un**-**patterned)areas of GaAs(100).** Strip patterns were etched along [01–1] (**a**). The height of strip is approximately 500 nm as clearly seen in the enlarged side-view SEM images in (**b**) and (**c**).

The strip patterns used in this experiment were fabricated using conventional photolithography technique and wet chemical etching. As clearly shown in Figure 1 (a), the strip patterns were fabricated on GaAs (100) along [01–1] and the width of strips are approximately 220 μm and of the trenches are approximately 70 μm. The height of the strip pattern is approximately 500 nm as clearly seen in Figure 1 (b, c), which are cutouts from the Figure 1 (a). The ‘strip top’ area was covered by photo-resist during the etching using a H_3_PO_4_:H_2_O_2_:H_2_O (3:1:100) solution while the ‘trench’ area was exposed. For the fabrication of Ga MDs on strip-patterned GaAs (100) surfaces, a Riber-32P solid-source MBE was used. To observe the substrate temperature (*T*_sub_) and growth rate (*G*_rate_) of surface reconstructions and growth procedures, an *insitu* reflection high-energy electron diffraction was utilized. For a consistent set of experiments, growth procedures were kept similarly between samples. After mounting the samples on molybdenum sample holder block (moly-block), it was degassed at the *T*_sub_ of 350°C for an hour. Then the moly-block was introduced in a main growth chamber through ultra-high vacuum transfer modules. The *T*_sub_ was then raised to 600°C by 50°C/min. Subsequently, by annealing substrates at the *T*_sub_ of 600°C for 10 min the native Ga surface oxide (Ga_2_O_3_) was removed. From our previous experiments on buffer growth on shallow patterned substrates, the buffer growth destroyed the pattern shapes (trenches were filled and sidewalls were smoothened) due to high anisotropic surface diffusion during the buffer growth [40,41]. Thus, a buffer layer was avoided in this experiment. After annealing the *T*_sub_ was lowered to 400°C for the fabrication of Ga MDs. For the consistency of the results and minimization of the arsenic monomer background, the chamber background pressure was kept below 4 × 10^−9^ Torr for each growth. The arsenic monomer background pressure was below 10^−12^ Torr under this pressure. Now based on an equivalent amount of GaAs growth with As_4_ flux, 20, 10, and 5 ML of Ga were deposited on strip-patterned GaAs (100) surfaces at the *T*_sub_ at 400°C to form metal Ga droplets. The *G*_rate_ used was 0.5 ML/s. Then, the *T*_sub_ was quenched down right after the fabrication in order to minimize Ostwald ripening [42,43]. An SEM under vacuum and AFM in air was used for the characterization of surface morphology [44-46]. Energy dispersive X-ray spectrometry (EDS) under vacuum was used for the chemical composition analysis and NanoScope (Bruker Corporation, Billerica, MA, USA), WSXM Nanotec Electronica S.L, Tres Cantos (Madrid) SPAIN [47] and Origin software (Origin Software Inc., San Clemente, CA, USA) were used for the analysis and processing of the acquired data.

## Results and discussion

**Figure 2:**
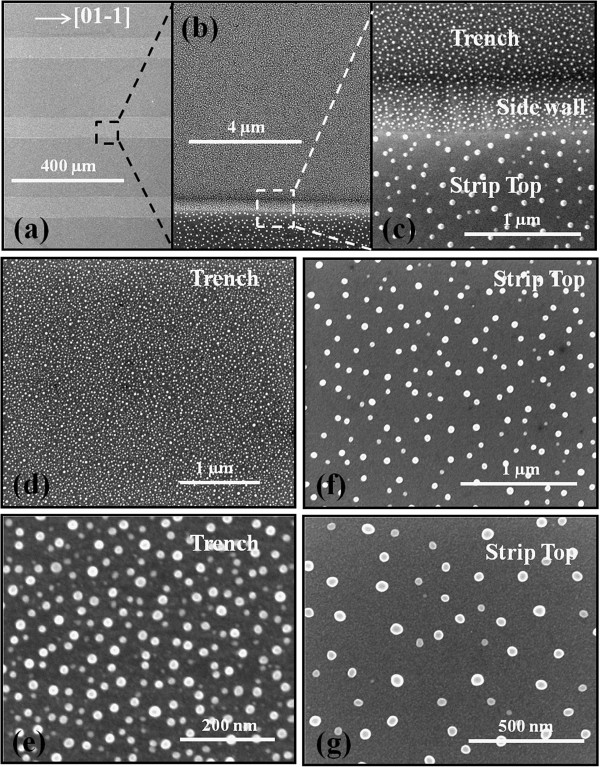
**SEM images of the interface between trench(patterned)and strip top(un**-**patterned)areas of GaAs(100).** SEM images of the interface between the trench (patterned) and strip top (un-patterned) areas of GaAs (100) showing the sharp contrast of size and density of Ga metal droplets. Ga droplets were fabricated with the deposition of 20 ML at the *T*_sub_ of 400°C.

**Figure 3:**
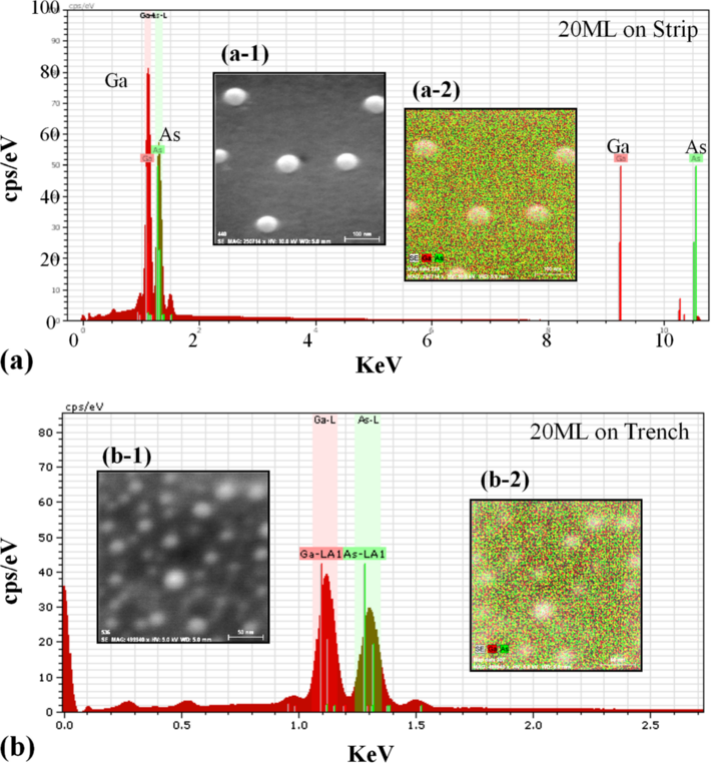
**EDS of Ga droplet samples with 20 ML deposition.** EDS of Ga droplet samples with 20 ML deposition on strip top area in (**a**) and trench area in (**b**). Insets show the SEM images (a-1) and (b-1) and the corresponding EDS mappings (a-2) and 3(b-2).

Figure 2 shows the sharp contrast of the density and size of Ga MDs at the interface between trench and strip top areas by the SEM images. The Ga MDs were fabricated with 20 ML at the surface temperature (*T*_sub_) of 400°C. Figure 2 (b) is a cutout from the Figure 2 (a) and similarly, Figure 2 (c) is from Figure 2 (b). With an enlarged view of Figure 2 (c) at the interface between etched and un-etched areas, the sharp contrast in size and density is clearly observed between the strip top and trench areas. In Figure 2, (d) and (f) are further enlarged images of trench areas and in the same way in Figure 2, (e) and (g) are from strip top areas. By comparing the strip top and trench areas, the density of Ga MDs is relatively higher and the size is much smaller on the trench area. Meanwhile, the density of MDs is much lower and the size is much larger on the strip top area. As the image size of Figure 2 (f) is almost twice as large as Figure 2 (g), the size of MDs on strip top area in Figure 2 (g) is indeed much larger. Figure 3 shows EDS analysis of Ga MD samples with 20 ML deposition on both strip top area in Figure 3a and trench area in Figure 3b. The EDS analysis confirmed the presence of elemental signal of Ga and As and the higher Ga peaks as expected. The SEM insets and EDS mappings show good matching and the MDs are indeed consisted of Ga as clearly shown in Figure 3 (a-2) and (b-2).

**Figure 4:**
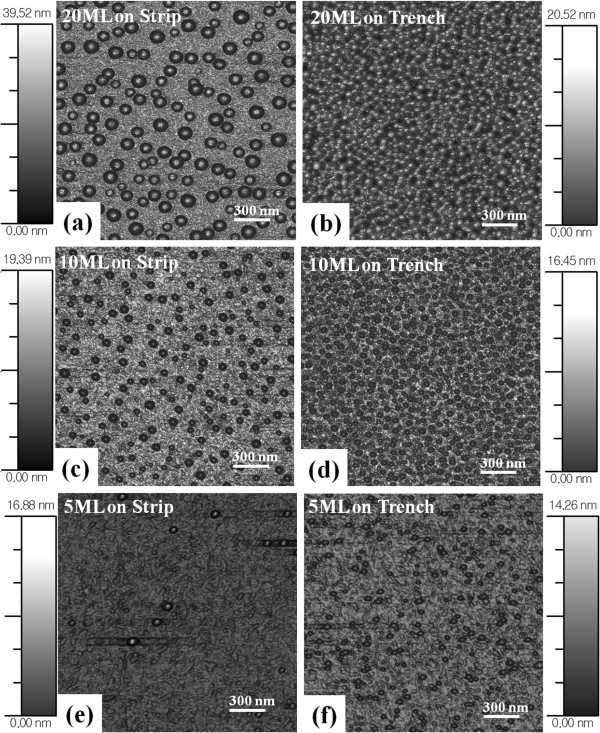
**3**-**D top**-**view atomic force microscope** (**AFM**) **images.** 3-D top-view atomic force microscope (AFM) images show the surface morphologies of Ga metal droplets on GaAs (100) with 20, 10, and 5-ML depositions at the *T*_sub_ of 400°C. AFM images are 2(*x*) × 2(*y*) μm^2^, and scale bars correspond to the heights of the images.

**Figure 5:**
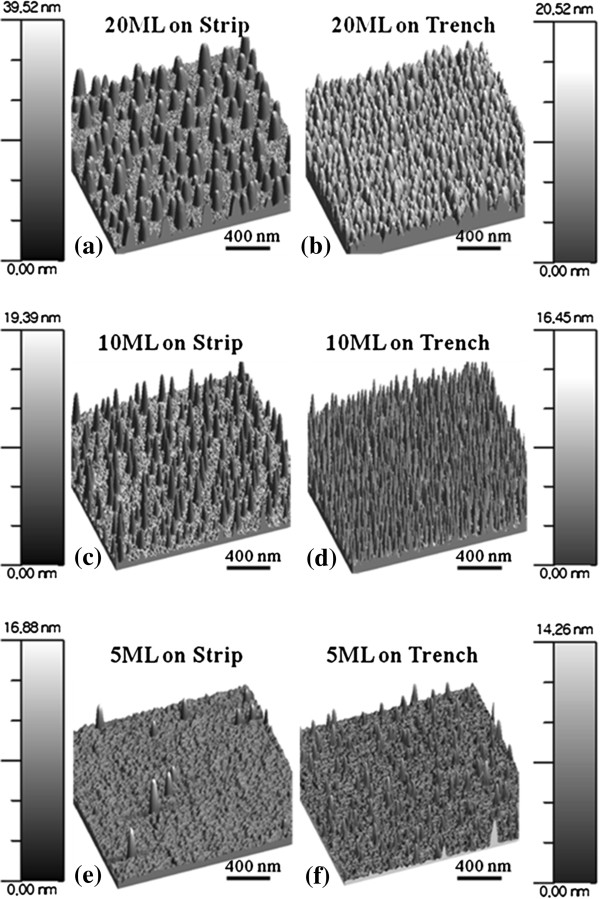
**3**-**D side**-**view AFM images of the variation of Ga metal droplets;20 in (a) and (b), 10 in (c) and (d), and 5-ML depositions in (e) and (f) at the *T***_**sub**_**of 400°C.** Figures correspond to the images in Figure 4: Figures 4a-5a, etc.

**Figure 6:**
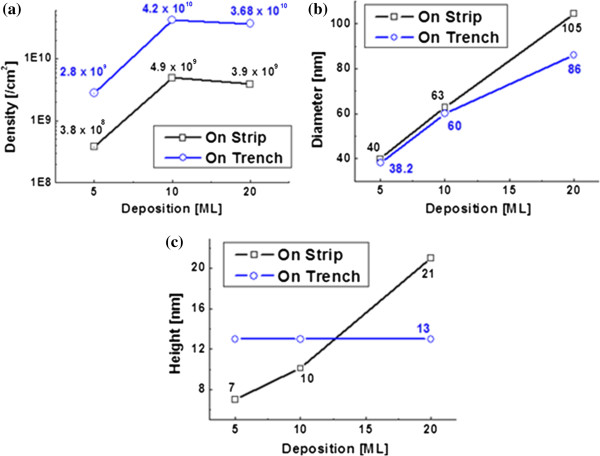
**Plots of density**, **diameter and height of Ga metal droplets with variation of ML deposition.** Data from the strip top and trench are plotted together per each deposition for a straightforward comparison.

A systematic variation of Ga ML deposition on strip-patterned GaAs (100) is demonstrated and shown in Figures 4 and 5; the summary plots of density, diameter, and height are shown in Figure 6. Figure 4 shows 2-D flat AFM views of Ga MD formation on the strip and trench areas with 20 ML in Figure 4a,b and with 10 ML in Figure 4c,d and 5 ML in Figure 4e,f. For the side-view perspective, Figure 5 shows the 3-D side AFM views of Ga MDs similarly with 20, 10, and 5 ML. With 5-ML deposition, the average density was 2.8 × 10^9^ cm^−2^ on trench area, while it was 3.8 × 10^8^ cm^−2^ on the strip area. There was about an order of magnitude difference between the strip and trench areas. With an increase of ML to 10, the density was increased to 4.2 × 10^10^ cm^−2^ on the trench and to 4.9 × 10^9^ cm^−2^ on strip. Also, there was about an order of difference between the two areas in the average density of Ga MDs. With a further increase of ML deposition to 20, the average density was slightly decreased to 3.68 × 10^10^ cm^−2^ on the trench area and to 3.9 × 10^9^ cm^−2^ on strip area. In previous experiments, an increase of the average MD density was observed when ML deposition was increased [36,37]. Also, slightly reduced density was observed depending on the growth conditions, i.e., duration, *G*_rate_, and *T*_sub_. Here the *T*_sub_ was fixed at 400°C and the *G*_rate_ was also fixed at 0.5 ML/s for all samples. Thus, the growth duration was increased with increased deposition amount. With 5-ML deposition, the MDs begun to nucleate, and the density and size were increased when ML deposition was increased to 10, reaching the peak density. With a further increase of ML to 20, which is equivalent to the duration of 40 s in this experiment, the MDs could have sufficient time to diffuse and merge. Once the merging of MDs occurs, bigger MDs tend to absorb the smaller ones and this process can result in a reduced density, which is known as Ostwald-ripening [42,43]. To minimize Ostwald-ripening, the duration has to be reduced but this requires a variation of the growth parameter, *G*_rate_ in this case. For the diameters of MDs, the average diameters were larger on strip top areas as clearly seen in Figure 6b as well as in the AFM images of Figures 4 and 5. Both the strip and trench pattern show increased average diameters when ML was increased. At 5 ML on strip, the diameter was 40 nm and increased to 63 nm with 10 ML and to 105 nm with 20 ML. On the trench areas, the average diameters of Ga MDs were 38.2 nm with 5 ML, 60 nm with 10 ML, and 86 nm with 20 ML. As the ML deposition was increased, the gap between the strip top and trench areas became larger perhaps due to Ostwald-ripening as discussed. The increased diameter of Ga MDs is a common trend with increase in deposition amount [36,37]. Now, for the average height of Ga MDs as seen in Figure 6c, on strip top areas, it showed a constant increase. An increased height of MDs is also an acceptable result when ML deposition is increased in conditions of atomically smooth surfaces. However, the height of MDs on trench areas kept almost the same regardless of the ML variation in this experiment. This indicates that the amount of deposition was dedicated either to the expansion of diameter or to the increase of density if there was no intermixing or desorption involved in the process [36,37]. Considering the *T*_sub_ of MD fabrication, we could exclude the intermixing and desorption to some degree. The diameter of Ga MDs in Figure 6b does not seem to be unusual, indicating the blue line stays below the black. The density of MDs on the trench area (blue line in Figure 6a) shows that the increased deposition was mostly used for the increase of density; the blue line stays above black. Also, this behavior can indicate that the surface is not atomically smooth on the trench area.

**Figure 7:**
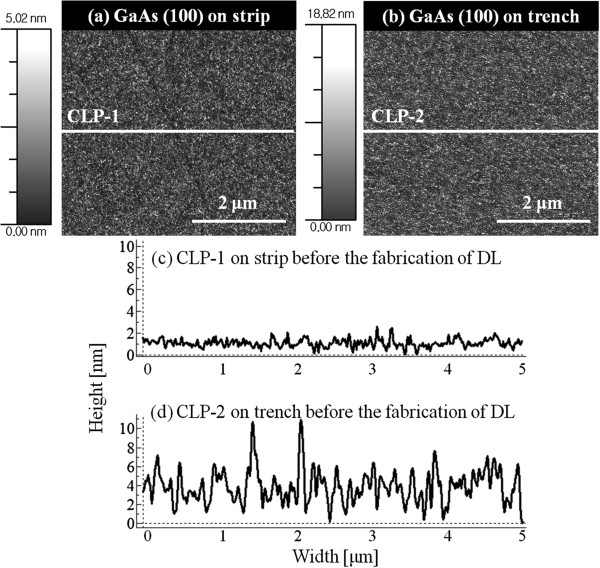
**AFM images of GaAs** (**100**) **on strip top and trench area before fabrication of Ga MDs.** AFM images of GaAs (100) on (**a**) strip top area and (**b**) trench area before the fabrication of Ga MDs. (**c**) and (**d**) show the cross-sectional line profiles (CLPs) of two areas. White lines in (a) and (b) are the corresponding locations of CLPs shown in (c) and (d).

Figure 7 shows the cross-sectional line profiles (CLPs) of bare GaAs (100) on strip area in Figure 7a and trench area in Figure 7b before Ga MD fabrication. Figure 7c is the CLP on the strip area and likewise, Figure 7d is on trench area shown as white lines in Figure 7a, b. The lengths of CLPs are 5 μm (*x*-axes on the graphs), and height was set at 10 nm for a straightforward comparison. As clearly seen in CLPs, the trench area is much rougher, confirming the previous speculation based on MD size analyses. The strip area showed an RMS roughness of 0.39, while the trench area was 2.26 which indicates that the trench area is approximately 5.8 times rougher. This large difference on the atomic surface roughness could be the major cause for the sharp contrast on the size and density of Ga MDs. A smoother surface can indicate a longer diffusion length and *vice versa*. When the diffusion length is longer, we generally observe lower density and larger size of MDs, for example, Ga and In MDs [36,37]. We can easily observe the effect of diffusion length when *T*_sub_ is increased and when other conditions were kept the same, as a higher *T*_sub_ indicates a longer diffusion length. For example, the MDs which are fabricated at 500°C as compared to 400°C should have larger dimensions and thus lower density and *vice versa*. In this experiment, the density is nearly an order of magnitude higher on strip areas almost constantly for 5, 10, and 20 ML. Also, the average diameters are larger on strip patterns as the surface is much smoother.

## Conclusions

In conclusion, the sharp contrast of the size and density of Ga MDs on photo-lithographically patterned GaAs (100) was demonstrated and clearly observed by SEM and AFM. The EDS analysis confirmed that the MDs were consisted of Ga atoms. Also a systematic control of size and density was demonstrated by ML variation, and the behavior was discussed with atomic surface roughness, diffusion length, and surface diffusion. Ga MDs were fabricated by solid-source MBE, and the density of MDs was generally higher on the trench areas, and the size was larger on strip tops due to the approximately 5.8 × smoother surface morphology.

## Competing interests

The authors declare that they have no competing interests.

## Authors’ contributions

JL, YH, SK participated in the experiment design and carried out the experiments. ML, MS, SS, EK JL participated in the analysis of data. GS, JL designed the experiments and testing methods. ML, JL carried out writing. All authors helped in drafting and read and approved the final manuscript.
